# Exploring the Passive Second Stage of Labour and Related Perinatal Outcomes in a Physiological Cohort

**DOI:** 10.1111/birt.70072

**Published:** 2026-05-02

**Authors:** Lucia Zagra, Maria Panzeri, Simona Fumagalli, Teresa Gramegna, Antonella Nespoli, Sara Ornaghi, Anna Locatelli

**Affiliations:** ^1^ Department of Obstetrics Fondazione IRCCS San Gerardo dei Tintori Monza Italy; ^2^ School of Medicine and Surgery University of Milano‐Bicocca Milano Italy

**Keywords:** healthy women, labor progression, normal birth, passive second stage of labor

## Abstract

**Background:**

The passive second stage of labor has been increasingly recognized as a normal phase of labor. However, it remains poorly studied, especially in healthy women. This study aims to assess the passive phase in healthy laboring women and maternal and neonatal outcomes.

**Methods:**

A retrospective cross‐sectional study on women who gave birth in an Italian University maternity center between 2019 and 2023. Only healthy laboring women without epidural analgesia or intrapartum interventions were included. Data were extracted from electronic records and analyzed overall and by parity.

**Results:**

A passive second stage was identified in 466 out of 2810 eligible women (16.6%), with a median duration of 30 min. The passive phase occurred more frequently in nulliparous women (63.9%; *p* < 0.001) and was associated with more advanced cervical dilatation at partograph initiation (*p* < 0.001) and longer second‐stage duration (*p* < 0.001), even when stratified by parity. Analysis stratified by parity showed higher rates of episiotomy (*p* = 0.02) and lithotomy position (*p* = 0.013) among nulliparas, and of neonatal macrosomia (*p* = 0.001) and NICU admissions (*p* = 0.009) among multiparas with a passive second stage. Mode of birth did not differ in either group.

**Discussion:**

The passive second stage occurred in a minority of healthy laboring women and may be underdiagnosed due to inconsistent definitions and reliance solely on vaginal examinations. Recognizing and accurately documenting this phase may contribute to a more precise description of physiological labor progression, without implying causal effects on maternal or neonatal outcomes.

## 1 Background

Labor can be conceptualized as a physiological continuum from pregnancy to the birth of the baby and placenta. There are no abrupt transitions between stages; instead, the phases of labor overlap, with shared physiological changes across them [1]. Labor progression is governed by a complex interplay of biological, emotional, and organizational factors [1, 2, 3]. Understanding this physiology is essential for healthcare providers to make informed decisions, safeguard maternal and neonatal well‐being, and support a positive birth experience [4, 5].

The second stage begins at full cervical dilatation and ends with the birth of the baby [1, 3, 5, 6]. Physiologically, this stage often includes a temporary decrease in uterine contractility following full dilatation but prior to the onset of active pushing. As the fetal head descends into the pelvis, the uterus becomes less distended, leading to a physiological lull characterized by less frequent and less intense contractions [3, 7]. This period may facilitate fetal rotation, maternal recovery, and reorganization of uterine muscle fibers before the effortful pushing phase. Moreover, the relative pause in contractile intensity may enhance utero‐placental perfusion and fetal oxygenation, potentially resulting in a different effect on fetal well‐being compared to the active pushing phase [8].

Characterizing the passive second stage of labor in healthy women provides important descriptive data on labor progression, which can help identify factors associated with maternal and neonatal outcomes [3]. The presence of a passive phase within the second stage, before involuntary or directed pushing, has gained increasing attention in both research and clinical contexts [3].

Despite growing recognition of its existence, the passive second stage of labor remains poorly defined in the scientific literature. Most studies do not distinguish this phase from the active second stage of labor or focus on different aspects without offering a clear definition. Research on the second stage has primarily handled the management strategies—particularly comparing immediate versus delayed pushing [9, 10, 11, 12, 13]—without clearly delineating the distinct phases within it.

Only a few recent studies have explicitly identified and explored the passive second stage of labor [8, 14, 15, 16, 17]. These investigations focused on associations between the duration—or prolonged duration—of the passive phase (using varying definitions) and antenatal or intrapartum factors, as well as maternal and neonatal outcomes. However, they have been limited to nulliparous women receiving epidural analgesia or other intrapartum interventions. Bjelke et al. [14] reported that a passive phase longer than four hours was associated with increased risks of caesarean section and operative vaginal birth, and lower maternal birth satisfaction, but showed no association with other maternal outcomes. Erickson et al. [16] found that a longer passive phase correlated with a longer duration of active pushing. Other identified risk factors for a prolonged passive phase (more than 2 h) include analgesia use, fetal malposition, and birthweight over 4000 g [8], while Collinot et al. [15] reported no association between a passive phase > 4 h and adverse maternal or neonatal outcomes.

Although the passive phase is gaining recognition, major guidelines differ in their definitions. The World Health Organization (WHO) [5], the American College of Obstetricians and Gynecologists (ACOG) [18] and the Royal Australian and New Zealand College of Obstetricians and Gynecologists (RANZCOG) [19] do not clearly define the passive second stage of labor, noting only that the urge to push may vary among women. In contrast, NICE explicitly defines the passive second stage of labor as the period of full cervical dilatation before or in the absence of involuntary or active pushing, and defines the onset of the active second stage as the presence of involuntary or active pushing with full dilatation or when the fetal head becomes visible [6]. Other guidelines also explicitly recognize the passive second stage. The Society of Obstetricians and Gynecologists of Canada (SOGC) [20] limits its duration to 2 h in nulliparous and 1 h in multiparous women. NICE allows up to 2 h for women with epidurals [6]. Queensland recommends reassessment after 1 h without progress or urge to push, regardless of parity or analgesia [21].

The lack of standardized terminology and definitions contributes to clinical ambiguity, potentially leading to the misinterpretation of physiological labor patterns as pathological dystocia [21]. This is particularly relevant in healthy women without epidural analgesia, where spontaneous labor may unfold naturally without pharmacological support. In such cases, failure to recognize the passive phase may lead to premature pushing, unnecessary interventions, and disruption of physiological processes.

To our knowledge, no studies have specifically investigated the prevalence or characteristics of the passive second stage of labor in healthy women without epidural analgesia or intrapartum interventions. Gaining insight into its physiology, prevalence, and associations with maternal and neonatal outcomes is crucial for informing evidence‐based, individualized care and avoiding overdiagnosis of dystocia in physiological labor.

The primary objective of this study was to estimate the prevalence of the passive second stage of labor in healthy laboring women. Secondary aims were to identify antenatal and intrapartum factors associated with its presence and to evaluate related maternal and neonatal outcomes.

## 2 Methods

### 2.1 Study Design

A retrospective cohort study was conducted to reach the study aims.

### 2.2 Setting

The study was conducted in the Maternity Unit of a university hospital in Northern Italy, which attends approximately 2500 births per year. The unit provides care for both healthy and high‐risk women, ensures one‐to‐one midwifery care, and promotes zero separation of the triad.

Upon admission to the birth suite, women are allocated to a specific model of care based on obstetric risk, in accordance with regional guidelines [22] and described in previous research conducted in the same setting [23]. A standardized checklist is used to assess maternal and fetal risk factors at the time of hospital admission. Healthy women are managed independently by midwives. Those with identified risk factors receive care within a shared model involving a multidisciplinary team composed of a midwife, an obstetrician, a dedicated anesthesiologist, and neonatal staff. All women are offered one‐to‐one midwifery care. The assigned midwife promotes, monitors, and supports labor progression by encouraging oral intake and free movement, assessing pain coping abilities, and promoting appropriate pain relief methods—whether pharmacological or non‐pharmacological—always taking into account the woman's preferences, fetal well‐being, and the progression of labor.

### 2.3 Participants

The study included women who gave birth between January 1, 2019, and December 31, 2023, and met the eligibility criteria defined by Regional Resolution No. 268/2018 of the Lombardy Region [22], which outlines the characteristics of healthy pregnant women suitable for a midwifery care model. The model described in this resolution supports the central role of midwives in providing autonomous, continuous care to healthy pregnant women, within an integrated multidisciplinary framework that ensures collaboration with obstetricians and other healthcare professionals when needed.

Eligible participants were healthy women with a singleton pregnancy in cephalic presentation, a gestational age between 37 + 0 and 41 + 6 weeks and an estimated fetal weight between 2500 and 4000 g. Additional inclusion criteria included maternal age between 18 and 45 years, absence of comorbidities (e.g., obesity, moderate anemia ‐Hb < 8.5 mg/dL‐ or thrombocytopenia at labor onset ‐< 100,000/μL‐, uncontrolled hypothyroidism, or substance use), and no fetal anomalies. Labor was required to begin spontaneously, with normal amniotic fluid volume, intact membranes or clear fluid in cases of premature rupture of membranes (PROM) lasting less than 24 h, no evidence of placenta previa or abnormal placentation with associated bleeding, and no history of uterine surgery or major obstetric complications.

Women were excluded if, during the first stage of labor, care was transferred to a shared model, based on clinical indications. Exclusion criteria also included the need for intrapartum interventions such as analgesia, oxytocin augmentation, or amniotomy, as well as the presence of abnormal fetal heart rate patterns, intrapartum bleeding, or meconium‐stained amniotic fluid.

### 2.4 Variables

The primary outcome of this study was the probability of experiencing a passive second stage of labor among healthy laboring women. To address this objective, we considered the number of women who experienced the passive phase, as recorded in the electronic clinical database.

For this study, the passive second stage of labor was defined according to the NICE intrapartum care guideline as the period of full cervical dilatation before or in the absence of involuntary or active pushing. The onset of the active second stage was defined as the presence of involuntary or active pushing with full cervical dilatation or when the fetal head became visible [6].

To investigate the secondary aim, we extracted the following variables from the data sources: socio‐demographic and obstetric characteristics, intrapartum factors, and maternal and neonatal outcomes. Socio‐demographic variables included maternal age and country of origin. Obstetric characteristics comprised parity and gestational age at birth (in weeks). Intrapartum variables encompassed cervical dilatation at partograph initiation, use of non‐pharmacological pain relief methods (maternal positions and water immersion), and the duration of each labor stage—specifically, the active phase of the first stage, the passive and active phases of the second stage, the total duration of the second stage and delayed second stage (also referred to as prolonged second stage), defined as a total second‐stage duration > 2 h, including both passive and active phases. The passive phase was considered a physiological subphase within the second stage, whereas delayed second stage reflects prolonged overall duration of the second stage.

Maternal outcomes included mode of birth (spontaneous vaginal birth, vacuum‐assisted vaginal birth, or caesarean section), birth position (specifically lithotomy), occiput posterior fetal position (OPP) at birth, episiotomy, severe perineal tears (defined as third‐ and fourth‐degree tears), and postpartum hemorrhage (defined as blood loss > 1000 mL). Neonatal outcomes included low Apgar score at 5 min (< 7), birth weight > 4500 g, and admission to the Neonatal Intensive Care Unit (NICU) during the hospital stay.

Parity was dichotomized as “nulliparous” versus “multiparous,” and further classified according to whether the woman had given birth three or more times (yes/no). Cervical dilatation at partograph initiation was analyzed both as a continuous variable and through two dichotomous categories: 4–6 cm (yes/no) and ≥ 9 cm (yes/no).

### 2.5 Data Sources

To achieve the study aims, records of all women who gave birth between January 1, 2019, and December 31, 2023, were retrospectively screened using the electronic birth register. This register, routinely completed for every woman who gives birth in the maternity unit, contains detailed information on socio‐demographic data, antenatal and intrapartum characteristics, postnatal outcomes, and the care provided throughout the childbirth process.

The electronic birth registry includes a standardized electronic partograph completed prospectively by the attending midwife. The partograph requires structured documentation of the time of full cervical dilatation and the onset of involuntary or active pushing, allowing the duration of the passive second stage of labor to be calculated as the interval between these two time points. Data entry is performed in real time as part of routine clinical care and is subject to institutional data quality checks.

### 2.6 Statistical Methods

Data analyses were conducted, and the manuscript was written in accordance with the *Strengthening the Reporting of Observational Studies in Epidemiology (STROBE)* guideline [24].

Descriptive statistics were calculated for baseline characteristics overall and stratified by the presence or absence of a passive phase in the second stage of labor. For continuous variables, distributions were visually assessed; means and standard deviations or medians and interquartile ranges (IQRs) were reported as appropriate. Group comparisons for continuous variables were performed using Student's *t*‐test and Mann–Whitney test as appropriate. For categorical variables, proportions were calculated and compared using Chi‐square tests. To reach the study aims, potential confounding due to the retrospective observational design was addressed. A stratified analysis by parity (nulliparous vs. multiparous) was performed to explore these associations more accurately.

All statistical tests were two‐sided, and a *p*‐value < 0.05 was considered statistically significant. Analyses were performed using RStudio software (version 4.3.2).

## 3 Results

Between January 1, 2019, and December 31, 2023, a total of 12,570 women gave birth in the Maternity Unit where the study was conducted. Of these, 26.9% (*n* = 3406) were healthy laboring women who gave birth during the study period. Among them, 596 women (17.5%) were excluded due to intrapartum interventions during the first stage of labor. The final study sample consisted of 2810 women (Figure 1). Of these, 2233 (79.5%) were of Italian origin, 282 (10.0%) were from other European countries, and 295 (10.5%) from non‐European countries. The cohort included 1140 nulliparous women (40.6%) and 1670 multiparous women (59.4%). The mean gestational age at birth was 39.41 weeks (±1.03). Sociodemographic and antenatal characteristics of the study population are summarized in Table 1.

**FIGURE 1:**
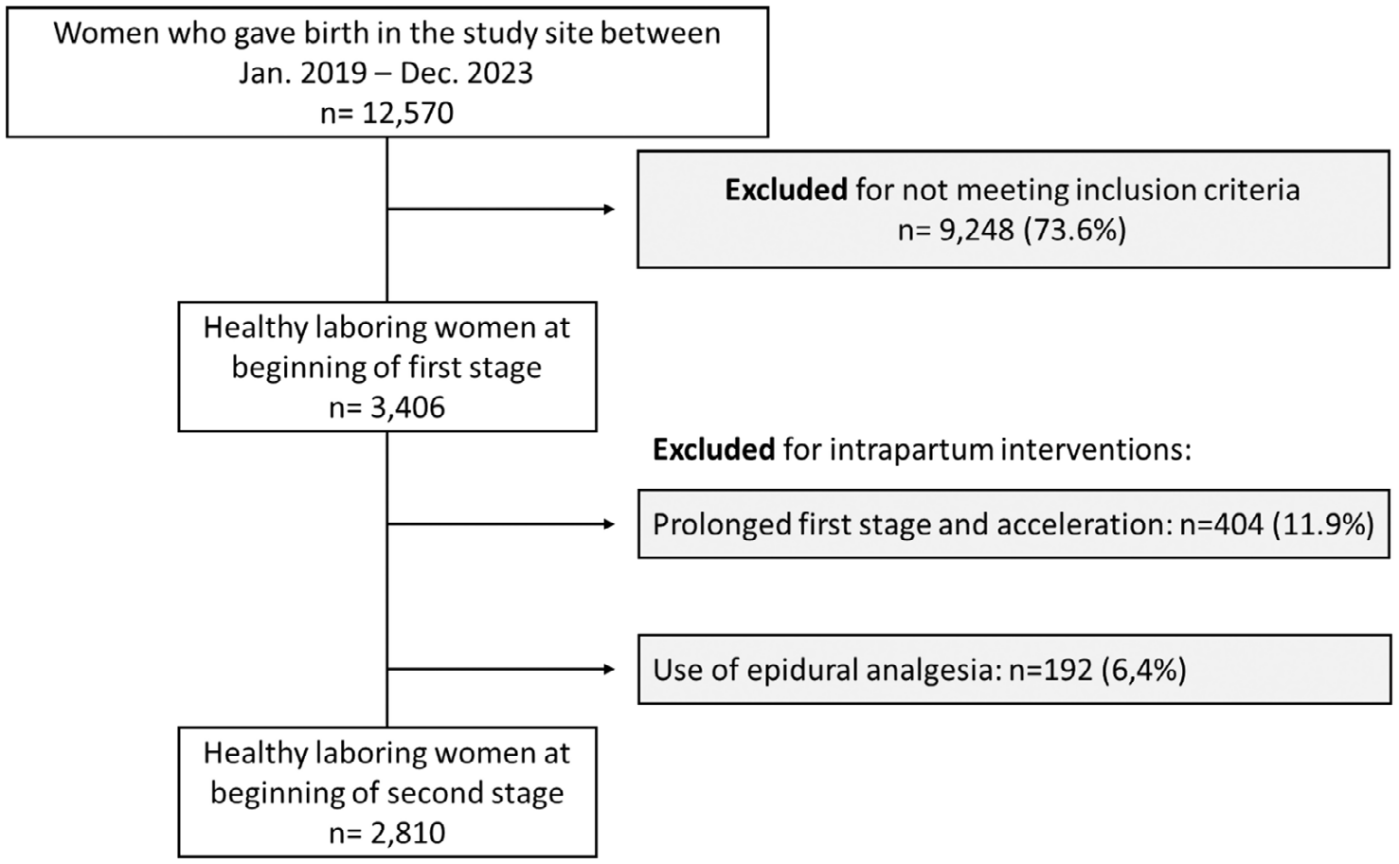
Flow diagram of the sample recruitment process.

**TABLE 1:** Distribution of socio‐demographic and obstetric characteristics by the occurrence of the passive phase of the second stage.

	Overall (*n* = 2810)		Passive phase no (*n* = 2344; 83.4%)		Passive phase yes (*n* = 466; 16.6%)		*p*
Age	32.2	±4.7	32.2	±4.7	32.4	±4.6	0.514
Country of origin							
Italy	2233	79.5%	1851	82.9%	382	17.1%	0.07
Other European countries	282	10.0%	233	82.6%	49	17.4%	
Non‐European countries	295	10.5%	260	88.1%	35	11.9%	
Nulliparous	1140	40.6%	842	73.9%	298	26.1%	< 0.001
Multiparous	1670	59.4%	1502	89.9%	168	10.1%	
Parity > 3	34	1.2%	31	9.12%	3	8.8%	0.321
Gestational age at birth (weeks)	39.4	±1.0	39.4	±1.0	39.5	±1.1	0.027
Birth weight > 4500 g	9	30.0%	4	44.4%	5	55.6%	0.007

*Note:* Percentages are calculated within rows.

Of the 2810 women included in the study, 466 (16.6%) experienced a passive second stage of labor, with a median duration of 30 min (IQR: 20–60). In the nulliparous group (*n* = 1140), 298 women (26.1%) experienced a passive phase, while in the multiparous group (*n* = 1670), 168 women (10.1%) experienced a passive phase (*p* < 0.001).

### 3.1 Antenatal Factors Associated With the Passive Phase

Among antenatal factors, parity, gestational age at birth, and fetal macrosomia (birthweight > 4500 g) were significantly associated with the likelihood of experiencing a passive second stage of labor (parity: *p* < 0.001; gestational age: *p* = 0.027; macrosomia: *p* = 0.007) (Table 1). Fetal macrosomia was considered as an antecedent factor, as fetal size precedes the passive phase. Nulliparous women were more likely to experience a passive phase, with a median duration of 45 min (IQR: 30–60) compared to 20 min (IQR: 10–30) in multiparous women. Although a higher gestational age was statistically associated with a slightly increased likelihood of experiencing a passive phase, the effect size was small and unlikely to be clinically meaningful. Other antenatal factors are summarized in Table 1 and were not significantly associated.

### 3.2 Intrapartum Factors Associated With the Passive Phase

Among intrapartum factors, cervical dilatation at the initiation of partograph significantly influenced the likelihood of experiencing a passive second stage of labor. In our center, following NICE guidelines, the active phase of labor is diagnosed at ≥ 4 cm cervical dilatation, so the partograph is initiated at admission when this threshold is reached. Women whose partograph was started at lower cervical dilatation (4–6 cm) were significantly less likely to experience a passive phase, while those whose partograph was started at ≥ 9 cm had a higher likelihood (*p*‐Value < 0.001) (Table 2). Furthermore, the presence of a passive second stage of labor was associated with a longer active pushing phase, a longer total duration of the second stage, and a higher likelihood of a delayed second stage (all *p* < 0.001) (Table 2). Thus, the presence of a passive phase was associated with a longer active pushing phase, in addition to a longer total duration of the second stage.

**TABLE 2:** Distribution of intrapartum factors, maternal and neonatal outcomes by the occurrence of passive phase of second stage in the study sample.

Intrapartum factors	Overall (*n* = 2810)		Passive phase no (*n* = 2344; 83.4%)		Passive phase yes (*n* = 466; 16.6%)		*p*
Cervical dilation at partograph initiation	6	[5.0, 8.0]	6	[5.0, 8.0]	7.5	[5.0, 10.0]	< 0.001
Cervical dilation 4–6 cm at partograph initiation	1498	54.5%	1297	86.6%	201	13.4%	< 0.001
Cervical dilation ≥ 9 cm at partograph initiation	624	22.7%	435	69.7%	189	30.3%	< 0.001
Use of non‐pharmacological pain strategies	2463	87.7%	2042	82.9%	421	17.1%	0.063
Use of postures	2014	71.7%	1678	83.3%	336	16.7%	0.865
Use of water	663	23.6%	543	81.9%	120	18.1%	0.254
Duration of I stage (min)	75	[25.0,150.0]	75	[30.0,140.0]	90	[0.0,180.0]	0.468
Duration of active phase of II stage (min)	21	[11.0,45.0]	20	[10.0,39.0]	39	[17.0,69.7]	< 0.001
Duration of total second stage (min)	24	[12.00,53.0]	20	[10.0,39.0]	77.5	[40.0,125.0]	< 0.001
Delayed II stage	102	3.6%	51	50%	51	50%	< 0.001
*Maternal outcomes*							
Mode of birth							
Spontaneous vaginal birth	2772	98.6%	2316	83.5%	456	16.5%	0.1
Vacuum assisted vaginal birth	29	1.0%	20	69%	9	31%	
Caesarean section	9	0.3%	8	88.9%	1	11.1%	
Lithotomic position at birth	1248	45.0%	1021	81.8%	227	18.2%	0.029
Fetal OPP at birth	49	1.7%	41	83.7%	8	16.3%	1
Episiotomy^a^	136	4.9%	95	69.9%	41	30.1%	< 0.001
Severe perineal tear	61	2.2%	49	80.3%	12	19.7%	0.633
Blood loss > 1000 mL	70	2.5%	54	77.1%	16	22.9%	0.205
*Neonatal outcomes*							
Low Apgar score at 5 min (< 7)	2	0.1%	2	100%	0	0.0%	1
Admission to NICU	26	0.9%	16	61.5%	10	38.5%	0.006

*Note:* Data are presented as absolute frequencies (*n*) and row percentages (%) for categorical variables, and as median and interquartile range [IQR] for continuous variables.

Abbreviations: NICU, neonalta intensive care unit; OPP, occiput posterior position.

^a^ Among vaginal births only.

These associations were generally consistent when stratified by parity (Tables 3 and 4). An exception was observed for the association between the passive phase and prolonged second stage, which was statistically significant only among nulliparous women (*p*‐value < 0.001) (Table 3). Among multiparous women, a shorter first stage was associated with a higher likelihood of experiencing a passive second stage of labor (*p*‐Value < 0.001) (Table 4).

**TABLE 3:** Distribution of intrapartum factors, maternal and neonatal outcomes by the occurrence of passive phase of second stage within nulliparous women.

Intrapartum factors	Overall (*n* = 1140)		Passive phase no (*n* = 842; 73.9%)		Passive phase yes (*n* = 298; 26.1%)		*p*
Cervical dilation at partograph initiation	6	[5.0, 8.0]	6	[5.0, 8.0]	7	[5.00, 10.00]	< 0.001
Cervical dilation 4–6 cm at partograph initiation	632	56.5%	487	59.0%	145	49.5%	0.006
Cervical dilation ≥ 9 cm at partograph initiation	236	21.1%	136	16.5%	100	34.1%	< 0.001
Use of non‐pharmacological pain strategies	1059	92.9%	778	92.4%	281	94.3%	0.335
Use of postures	776	68.1%	565	67.1%	211	70.8%	0.269
Use of water	440	38.6%	338	40.1%	102	34.2%	0.083
Duration of I stage (min)	120	[60.0, 205.0]	120	[60.0, 195.0]	120	[0.0, 233.7]	0.676
Duration of active phase of II stage (min)	50	[30.0, 74.0]	47	[30.0, 69.0]	60	[34.0, 88.0]	< 0.001
Duration of total second stage (min)	58	[33.7, 91.0]	47	[30.0, 69.0]	110	[70.0, 152.7]	< 0.001
Delayed II stage	79	6.9%	31	3.7%	48	16.1%	< 0.001
*Maternal outcomes*							
Mode of birth							
Spontaneous vaginal birth	1115	97.8%	826	98.1%	289	97.0%	0.245
Vacuum assisted vaginal birth	19	1.7%	11	1.3%	8	2.7%	
Caesarean section	6	0.5%	5	0.6%	1	0.3%	
Litotomic position at birth	488	43.8%	343	41.5%	145	50.2%	0.013
Fetal OPP at birth	19	1.7%	14	1.7%	5	1.7%	1
Episiotomy^a^	105	9.3%	67	8.0%	38	12.8%	0.02
Severe perineal tear	49	4.3%	39	4.7%	10	3.4%	0.438
Blood loss > 1000 mL	30	2.6%	17	2.0%	13	4.4%	0.05
*Neonatal outcomes*							
Low Apgar score at 5 min (< 7)	0	0.0%	0	0.0%	0	0.0%	N/A
Admission to NICU	16	1.4%	10	1.2%	6	2.0%	0.45

*Note:* Data are presented as absolute frequencies (*n*) and row percentages (%) for categorical variables, and as median and interquartile range [IQR] for continuous variables.

Abbreviations: NICU, neonalta intensive care unit; OPP, occiput posterior position.

^a^ Among vaginal births only.

**TABLE 4:** Distribution of intrapartum factors, maternal and neonatal outcomes by the occurrence of passive phase of second stage within multiparous women.

Intrapartum factors	Overall (*n* = 1670)		Passive phase no (*n* = 1502; 89.9%)		Passive phase yes (*n* = 168; 10.1%)		*p*
Cervical dilation at partograph initiation	6	[5.0, 8.0]	6	[5.0, 8.0]	9	[5.50, 10.00]	< 0.001
Cervical dilation 4–6 cm at partograph initiation	866	53.1%	810	55.3%	56	33.9%	< 0.001
Cervical dilation ≥ 9 cm at partograph initiation	388	23.8%	299	20.4%	89	53.9%	< 0.001
Use of non‐pharmacological pain strategies	1404	84.1%	1264	84.2%	140	83.3%	0.869
Use of postures	1238	74.1%	1113	74.1%	125	74.4%	1
Use of water	223	13.4%	205	13.6%	18	10.7%	0.347
Duration of I stage (min)	55	[20.0, 112.2]	60	[20.7, 115.0]	17.5	[00.0, 105.0]	< 0.001
Duration of active phase of II stage (min)	13	[9.0, 21.0]	13	[9.0, 21.0]	15.5	[10.0, 25.0]	0.016
Duration of total second stage (min)	15	[9.0, 24.0]	13	[9.0, 21.0]	36	[25.0, 56.0]	0.001
Delayed II stage	23	1.4%	20	1.3%	3	1.8%	0.897
*Maternal outcomes*							
Mode of birth							
Spontaneous vaginal birth	1657	99.2%	1490	99.2%	167	99.4%	0.845
Vacuum assisted vaginal birth	10	0.6%	9	0.6%	1	0.6%	
Caesarean section	3	0.2%	3	0.2%	0	0.0%	
Lithotomic position at birth	760	45.9%	678	45.5%	82	49.1%	0.422
Fetal OPP at birth	30	1.8%	27	1.8%	3	1.8%	1
Episiotomy^a^	31	1.9%	28	1.9%	3	1.8%	0.897
Severe perineal tear	12	0.7%	10	0.7%	2	1.2%	0.78
Blood loss > 1000 mL	40	2.4%	37	2.5%	3	1.8%	0.78
*Neonatal outcomes*							
Low Apgar score at 5 min (< 7)	2	0.1%	2	0.1%	0	0.0%	1
Admission to NICU	10	0.6%	6	0.4%	4	2.4%	0.009

*Note:* Data are presented as absolute frequencies (*n*) and row percentages (%) for categorical variables, and as median and interquartile range [IQR] for continuous variables.

Abbreviations: NICU, neonalta intensive care unit; OPP, occiput posterior position.

^a^ Among vaginal births only.

### 3.3 Maternal and Neonatal Outcomes Associated With the Passive Phase

The association between the presence of a passive second stage of labor and maternal outcomes was explored. Experiencing a passive phase was significantly associated with a higher likelihood of giving birth in the lithotomy position (*p*‐Value 0.029) and undergoing an episiotomy (*p*‐Value < 0.001). No significant association was found with mode of birth (*p*‐Value 0.1).

Regarding neonatal outcomes, the overall number of events was low. While a higher rate of NICU admission was observed among women experiencing a passive phase (*p* = 0.013), the study is not adequately powered to assess neonatal outcomes, and these results should be interpreted with caution (Table 2).

When stratified by parity, among nulliparous women only maternal outcomes remained significant (Table 3), whereas among multiparous women only neonatal outcomes were observed (Table 4).

## 4 Discussion

To our knowledge, this is the first study to provide a quantitative estimate of the prevalence and duration of the passive second stage of labor in healthy women without epidural analgesia or intrapartum interventions, leading to a description of its physiological patterns. Additionally, we investigated antenatal and intrapartum factors associated with its occurrence, as well as related maternal and neonatal outcomes.

We observed that only 16.6% of women experienced a passive second stage of labor, with a higher prevalence among nulliparous women. The relatively low prevalence observed raises the possibility of underdiagnosis, potentially due to challenges in recognizing the passive phase, particularly in women with rapid labor progression. The NICE definition of the passive second stage, which distinguishes the period of full cervical dilatation without pushing from the active pushing phase, guided clinical documentation in our setting and informed the operational definition used in this study. Historically, diagnosis has relied solely on vaginal examination [6], which may limit detection—especially among multiparous women whose second stage often progresses quickly. It is plausible that the typically longer duration of labor in nulliparas may provide to midwives more time and clinical cues to recognize and document the signs of full cervical dilatation that facilitate the diagnosis of the passive phase [5, 6, 25].

Recent updates in the NICE guidelines [6] propose a more comprehensive diagnostic approach, allowing for the recognition of full dilatation through external clinical signs, with or without vaginal examination. However, during the study period (2019–2023), the earlier version of the NICE guidelines—based primarily on vaginal assessment—was still in use [26]. Furthermore, the lack of a precise WHO definition for the onset of the second stage may have contributed to inconsistencies in identification and documentation across clinical settings [5].

Regarding duration, we observed a median passive phase length of 30 min, with a significantly longer duration in nulliparous compared to multiparous women. Importantly, these durations are well within the physiological range defined by international guidelines and are markedly lower than the commonly used cut‐off values reported in the literature, even after stratification by parity [6, 20, 27, 28].

Among intrapartum factors, we observed that initiation of the partograph at an advanced cervical dilation (≥ 9 cm) was associated with a higher likelihood of experiencing a passive second stage of labor, regardless of parity. Conversely, early initiation (at 4–6 cm) was associated with a lower likelihood. These findings suggest that the trajectory of the first stage of labor may influence the occurrence of a passive phase. It is hypothesized that a rapid first stage might necessitate a brief physiological pause—a time for uterine reorganization—before the onset of effective pushing. Although this hypothesis is supported by qualitative studies describing midwives' practice of allowing a rest period after a swift first stage, our data do not directly address this aspect [29].

In both parity groups, the presence of a passive second stage of labor was associated with longer durations of the active phase of the second stage. The presence of a passive phase was associated with a longer duration of the active second stage and a longer total duration of the second stage, even after stratification by parity. Among nulliparous women, the passive phase was also associated with a delayed second stage, whereas this association was not observed among multiparous women. These results contrast with some previous studies focusing on the management of the second stage, which reported either a shorter active phase in the presence of delayed pushing [9, 10, 13, 30], or no difference at all [12, 31]. A likely explanation is the difference in study aim and populations studied: Previous research primarily investigated the effect of immediate versus delayed pushing, involving women who received epidural analgesia or other intrapartum interventions—factors that might influence labor duration and dynamics [6]. Our findings are consistent with the broader literature in associating the passive phase among nulliparas with a delayed second stage of labor [9, 10, 11, 12, 30].

In nulliparous women, the passive second stage of labor was also associated with a higher likelihood of giving birth in the lithotomy position and receiving an episiotomy. This reflects maternal outcomes observed in our cohort and highlights the importance of careful monitoring, without implying that the passive phase itself is protective or inherently safer [32].

Although we observed an association between the passive second stage of labor and increased NICU admissions, the overall number of events was low, and the causes for admission were heterogeneous and not consistently attributable to the passive phase itself. Overall, existing literature does not support a direct association between the passive second stage of labor and neonatal morbidity or NICU admission [15].

### 4.1 Strengths and Limitations

A major strength of this study is the inclusion of a strictly physiological cohort—women who experienced spontaneous labor and birth without epidural analgesia or other intrapartum interventions. By excluding factors known to interfere with the natural course of labor, this study offers a unique opportunity to observe the passive second stage of labor in its physiological progression. The large sample size and inclusion of both nulliparous and multiparous women enhance the generalizability of the findings. The study was conducted in a maternity care setting where midwifery‐led, one‐to‐one care during labor is standard practice and supported by organizational protocols that actively promote and protect physiological childbirth. While these features represent a favorable context for preserving physiological labor processes, they also constitute a potential source of variability, as organizational and institutional factors may influence how labor unfolds and how it is documented.

Limitations include the retrospective design and reliance on vaginal examinations for the documentation of labor stages, which may have led to underestimation of the passive phase. Although the electronic partograph includes structured fields for the timing of full cervical dilatation and onset of pushing, identification of the passive phase relied on clinical documentation and may be subject to misclassification or underdiagnosis, particularly in multiparous women with rapid labor progression. In addition, the exclusion of a substantial proportion of women due to intrapartum interventions may have introduced selection bias. This approach allowed us to focus specifically on physiological labor patterns, which was consistent with the aim of the study. However, it may have influenced the observed duration of labor and the occurrence of the passive second stage of labor, and therefore the findings are primarily generalizable to healthy women experiencing low‐risk, non‐interventionist labor. Moreover, the study was not adequately powered to assess neonatal outcomes due to the low number of events, and these results should be interpreted with caution. Furthermore, unmeasured variables such as midwives' clinical decision‐making, documentation practices, and local care models may have introduced inconsistencies in data capture. Finally, the definition of the passive second stage of labor was based on the NICE guidelines, which consider it the period of full cervical dilatation before active pushing, and this operational definition guided clinical documentation in our setting.

Future prospective studies involving physiological labors and integrating clinical signs, vaginal examinations, and women's subjective experiences could deepen our understanding of this clinically relevant labor phase.

## 5 Conclusion

This study provides a detailed characterization of the passive second stage of labor, including its prevalence, associated antenatal and intrapartum factors, and maternal outcomes. Among nulliparous women, the passive second stage of labor was associated with higher rates of episiotomy and lithotomy position, while gestational age, parity, and fetal macrosomia were identified as antecedent factors.

By fostering awareness and training on the passive phase second stage of labor, maternity care providers can better align care with physiological principles, potentially supporting more individualized and respectful management of labor.

These findings contribute to a clearer characterization of the passive second stage of labor as part of physiological labor progression. Our study provides novel evidence on its prevalence, duration, and associated factors in a low‐risk population, while highlighting the lack of clear associations with improved maternal or neonatal outcomes. Further research is needed to clarify its clinical significance, including the balance between potential risks and benefits and the impact of different second‐stage management approaches.

## Funding

The authors have nothing to report.

## Ethics Statement

The study was performed in line with the principles of the Declaration of Helsinki. Approval was granted by the Ethical Review Board of the ASST Vimercate (Approval Number: 37/2018).

## Conflicts of Interest

The authors declare no conflicts of interest.

## Data Availability

The data that support the findings of this study are available on request from the corresponding author. The data are not publicly available due to privacy or ethical restrictions.
